# Genomic Reporter Constructs to Monitor Pathway-Specific Repair of DNA Double-Strand Breaks

**DOI:** 10.3389/fgene.2021.809832

**Published:** 2022-02-14

**Authors:** Bert van de Kooij, Haico van Attikum

**Affiliations:** Department of Human Genetics, Leiden University Medical Center, Leiden, Netherlands

**Keywords:** genomic reporter constructs, double-strand break repair pathway choice, homologous recombination, end-joining, single-strand annealing

## Abstract

Repair of DNA Double-Strand Breaks (DSBs) can be error-free or highly mutagenic, depending on which of multiple mechanistically distinct pathways repairs the break. Hence, DSB-repair pathway choice directly affects genome integrity, and it is therefore of interest to understand the parameters that direct repair towards a specific pathway. This has been intensively studied using genomic reporter constructs, in which repair of a site-specific DSB by the pathway of interest generates a quantifiable phenotype, generally the expression of a fluorescent protein. The current developments in genome editing with targetable nucleases like Cas9 have increased reporter usage and accelerated the generation of novel reporter constructs. Considering these recent advances, this review will discuss and compare the available DSB-repair pathway reporters, provide essential considerations to guide reporter choice, and give an outlook on potential future developments.

## Introduction

**FIGURE 1:**
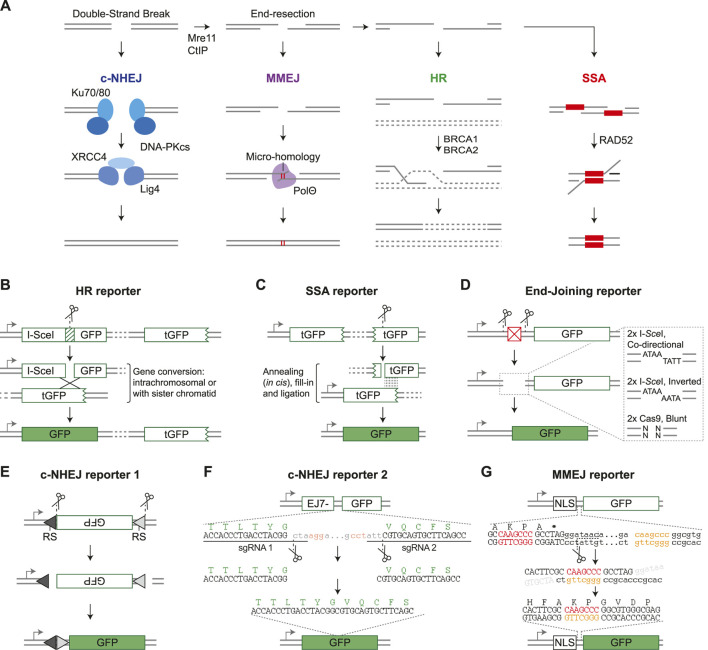
Fluorescent DSB-repair reporter systems. **(A)** Schematic diagram of the four double-strand break repair pathways and their main components. See main text for details. **(B)** DR-GFP (Pierce et al., 1999) is shown as an example HR reporter. Arrow = promoter, I-*Sce*I GFP is a GFP gene disrupted by insertion of an I-*Sce*I target site, tGFP = truncated GFP. **(C)** SA-GFP (Stark et al., 2004) is shown as an example SSA reporter. **(D)** Structure of a generic End-Joining reporter. Expression of GFP is prevented by a sequence element between the gene and the promoter (red box), but can be restored by nuclease-induced excision of this element followed by end-joining mediated repair of the distal DSB ends. The dashed-line box shows a zoom of the DSB ends which can be ligation compatible or non-compatible, depending on the nature of the nuclease and orientation of the target site. **(E)** Structure of pMX-INV (Bredemeyer et al., 2006), which is a VDJ recombination based c-NHEJ reporter specifically used in B-cells. It is cut by RAG nucleases, RS = Recombination Signal. **(F)** Structure of EJ7-GFP (Bhargava et al., 2018), which reports on error-free c-NHEJ. GFP amino acid sequence is depicted in green, sgRNA PAM sequences in red. Dots indicate omitted sequence. **(G)** EJ2-GFP (Bennardo et al., 2008) is shown as an example MMEJ reporter. Designed microhomology sequences are shown in red and orange, dots indicate omitted sequence.

The integrity of our genome is constantly challenged by DNA damaging lesions that arise during normal cell growth and division, and are caused by exposure to environmental mutagens and irradiation (Hoeijmakers, 2009). A particularly toxic lesion is the DNA Double-Strand Break (DSB), which separates a chromosome into two pieces and can thus cause detrimental karyotypic alterations. Detection of a DSB initiates an elaborate signaling response that halts the cell cycle, re-shapes the chromatin environment and recruits repair factors (Smeenk and van Attikum, 2013; Dantuma and van Attikum, 2016). The subsequent repair is performed by one of multiple repair pathways that are mechanistically distinct and range from error-free to highly mutagenic (Figure 1A; Scully et al., 2019).

In human cells the majority of DSBs are repaired by classical Non-Homologous End-Joining (c-NHEJ), which requires no or very little (≤4 nucleotides) homology at the DSB-ends to ligate them together (Figure 1A; Pannunzio et al., 2018). Repair by c-NHEJ can be either error-free, or introduce small insertions or deletions (InDels) at the break junction due to DSB end-processing by nucleases and polymerases. Alternatively, DSBs can be repaired by Homologous Recombination (HR), which is initiated by extensive nuclease-mediated resection of the DSB-ends to generate 3’ single strand overhangs (Figure 1A; Jasin and Rothstein, 2013). These overhangs invade homologous double-stranded DNA and prime polymerase-mediated extension. HR can then progress via several sub-pathways, as discussed in detail elsewhere in this special issue (Elbakry and Löbrich, 2021). In the dominant sub-pathway, the extended overhang anneals to the opposite DSB-end, and remaining single-stranded (ss) DNA gaps are closed. The genetically identical sister chromatid is the preferred repair template, and therefore HR is considered a high-fidelity repair pathway. However, other homologous sequences, either on nearby chromosomal DNA or on ectopically provided DNA, can also be used as repair template, even if homology is incomplete. In the latter case, HR can lead to sequence alterations at the repaired locus, which is known as gene conversion.

In addition to HR, DSB end-resection can prime repair by either Single-Strand Annealing (SSA) or by Micro-Homology Mediated End-joining (MMEJ), which is also known as alternative End-Joining or Theta-mediated End-joining (Figure 1A; Bhargava et al., 2016; Sallmyr and Tomkinson, 2018; Schimmel et al., 2019). During repair by both pathways, the opposite DSB-ends are joined by annealing of homologous sequence stretches, followed by nuclease-mediated removal of the non-homologous ssDNA ends. MMEJ requires short regions of microhomology (<20 nucleotides) adjacent to the DSB ends, whereas SSA depends on homologous repeats of at least 50 nucleotides long and can occur even if these are separated by distances up to 28 kilobases (Mendez-Dorantes et al., 2018; Kelso et al., 2019). Both pathways are mutagenic, but particularly SSA can result in large deletions.

Which pathway is employed to repair a given DSB is dependent on many factors including sequence context, chromatin environment, and cell cycle stage (Scully et al., 2019). Engagement of the appropriate repair pathway is essential for efficient genome maintenance, whereas an imbalance in pathway choice can have pathological consequences, including cancer development (Knijnenburg et al., 2018). This knowledge has driven studies on the fundamentals of DSB-repair for decades, but recently this research interest has grown exponentially due to the development of genome editing approaches using Cas9 and Cas9-like nucleases (Komor et al., 2017; Knott and Doudna, 2018). Understanding the determinants that direct repair of a Cas9-induced DSB towards a specific pathway is crucial to predict editing outcome, and to identify methods to control this outcome (Yeh et al., 2019). Notably, advances in genome editing techniques have not only spiked DSB-repair research, but also stimulated the development of methods to study it. This includes genomic DSB-repair reporter constructs, which have been essential tools in DSB-repair research by providing an easy and quantitative read-out for repair pathway activity (Gunn and Stark, 2012). Given these new developments, this review will discuss and compare the traditional and more recently published genomic DSB-repair reporter constructs.

## Single Pathway DSB-Repair Reporters

There are numerous published methods that could be considered reporter assays because they quantitatively detect DSB-repair by a given pathway. For simplicity, this review will be limited to describing the genomic reporter constructs that are designed to detect gain or loss of expression of a marker gene, as a result of defined sequence changes associated with DSB-repair by a specific pathway. The marker gene in the reporter could be a drug resistance cassette, and we will mention a few examples of such reporters. However, the main focus of this review will be on reporters that carry a marker gene that encodes a fluorescent protein (Table 1).

**TABLE 1:** Overview of fluorescent DSB-repair reporters. HR = Homologous Recombination, SSA = Single-Strand Annealing, c-NHEJ = classical Non-Homologous End-Joining, MMEJ = Microhomology-Mediated End-Joining, fs = frameshift, HITI = Homology Independent Targeted Integration, CD = Co-Directional orientation, Inv = Inverted orientation. “HR (templated)” indicates the requirement of an ectopically delivered repair template to detect repair by HR.

**Reporter**	**Nuclease**	**# of pathways**	**Pathway(s) analyzed**	**Reference**
DR-GFP	I-*Sce*I	1	HR	Pierce et al. (1999)
HR-Reporter	I-*Sce*I (2x, Inv)	1	HR	Mao et al. (2007)
pGC	I-*Sce*I	1	HR	Mansour et al. (2008)
SA-GFP	I-*Sce*I	1	SSA	Stark et al. (2004)
RMD-GFP	Cas9 (2x)	1	SSA	Mendez-Dorantes et al. (2018)
NHEJ-C	I-*Sce*I (2x, CD)	1	All distal end-joining	Seluanov et al. (2004)
EJ5-GFP	I-*Sce*I (2x, CD)	1	All distal end-joining	Bennardo et al. (2008)
sGEJ	I-*Sce*I (2x, CD)	1	All distal end-joining	Xie et al. (2009)
pEJ2	I-*Sce*I (2x, CD)	1	All distal end-joining	Mansour et al. (2010)
EJ6-GFP	Cas9 (2x)	1	All distal end-joining	Bhargava et al. (2017)
NHEJ-I	I-*Sce*I (2x, Inv)	1	Mutagenic distal end-joining	Seluanov et al. (2004)
pEJ	I-*Sce*I (2x, Inv)	1	Mutagenic distal end-joining	Mansour et al. (2008)
vGEJ	I-*Sce*I (2x, Inv)	1	Mutagenic distal end-joining	Xie et al. (2009)
EJ-RFP	I-*Sce*I	1	Mutagenic distal end-joining	Bindra et al. (2013)
pMX-INV	RAG (2x)	1	Error-free distal c-NHEJ	Bredemeyer et al. (2006)
EJ7-GFP	Cas9 (2x)	1	c-NHEJ	Bhargava et al. (2018)
EJ2-GFP	I-*Sce*I	1	MMEJ	Bennardo et al. (2008)
EJ7-GFP mHOM^a^	Cas9 (2x)	1	MMEJ	Bhargava et al. (2018)
Traffic Light Reporter (TLR)	I-*Sce*I	2	2 bp fs mutagenic end-joining + HR (templated)	Certo et al. (2011)
GFP to BFP conversion	Cas9	2	Mutagenic end-joining + HR (templated)	Glaser et al. (2016)
DNA repair reporter Arnoult	I-*Sce*I (2x, Inv)	2	Mutagenic distal end-joining + HR (templated)	Arnoult et al. (2017)
FIVER	Cas9 (2x)	2	All distal end-joining + HR/HITI (templated)^b^	Tennant et al. (2020)
CDDR (one cut variant)	Cas9	2	Mutagenic end-joining + HR (templated)	Eki et al. (2020)
HR-NHEJ Reporter	I-*Sce*I (2x, Inv)	2	Mutagenic distal end-joining + HR	Chen et al. (2019)
DSB-Spectrum_V1	Cas9 (2x)	2	Error-free distal c-NHEJ + HR	van de Kooij et al. (2021)
DSB-Spectrum_V2	Cas9	2	Mutagenic end-joining/SSA^c^ + HR	van de Kooij et al. (2021)
RFP-SCR	I-*Sce*I	2	Gene Conversion, Short Tract + Long Tract	Chandramouly et al. (2013)
SeeSaw Reporter	I-*Sce*I	2	>39 bp deletions^d^ + SSA	Gomez-Cabello et al. (2013)
CAT-R	Cas9 (2x)	2	Mutagenic end-joining + Large deletions^e^	Roidos et al. (2020)
CDDR (two cut variant)	Cas9 (2x)	2	Mutagenic end-joining + Error-free distal c-NHEJ	Eki et al. (2020)
SSA-TLR	I-*Sce*I	3	2 bp fs mutagenic end-joining + SSA + HR (templated)	Kuhar et al. (2016)
DSB-Spectrum_V3	Cas9	3	Mutagenic end-joining + SSA + HR	van de Kooij et al. (2021)

a Several variants of EJ7-GFP were constructed that contain 1–4 nucleotides microhomology.

b Either HR or HITI can be studied, depending on the provided repair template.

c Loss of BFP expression can result from mutagenic repair by either end-joining or SSA.

d The I-*Sce*I target site is located 39 bp behind the GFP sequence, so only repair resulting in deletions >39 bp will disrupt GFP expression.

e Which repair pathway is responsible for the large deletions that are detected by the CAT-R system has not been determined.

Early reporter systems were designed to study HR and the lay-out was based on the *MAT* locus of *Saccharomyces cerevisiae.* This locus is targeted by the HO endonuclease which can result in mating-type switching if the DSB is repaired by HR-mediated gene conversion using one of two homologous *HM* genes (Haber, 2012). To study this gene conversion process further, HR-reporter constructs were cloned that resembled the *MAT* locus, but contained marker genes like LacZ rather than the *MAT* gene (Rudin et al., 1989). This prototypic HR-reporter design was transferred to mammalian cells by the Jasin lab, which modified it to contain the target site for the I-*Sce*I nuclease rather than the HO nuclease, and GFP genes rather than LacZ genes (Rouet et al., 1994; Pierce et al., 1999). The resulting HR-reporter DR-GFP consists of two non-functional GFP gene repeats; the first is disrupted by insertion of an *I-Sce*I target site, while the second lacks a promoter and is C-terminally truncated (Figure 1B; Pierce et al., 1999). Expression of GFP, which can be measured by flow cytometry, is therefore dependent on I-*Sce*I-induced gene conversion between the two repeats, and serves as a quantitative read-out for HR. DR-GFP is currently still widely used and inspired the design of many other fluorescent DSB-repair reporter constructs (Table 1).

Following DR-GFP, Jasin *et al.* developed SA-GFP, an SSA-reporter that contains a C-terminally truncated GFP gene, and a second N-terminally truncated GFP gene with an I-*Sce*I site (Figure 1C; Stark et al., 2004). There is substantial sequence overlap between the truncated GFP genes, and annealing of these homologous sequences during SSA-repair of the I-*Sce*I induced DSB will generate an intact GFP gene. A limited number of alternative fluorescent HR and SSA reporters has been published (Table 1), which generally follow the same design principles as DR-GFP and SA-GFP, respectively.

In contrast to HR and SSA, there is an abundance of reporters to study repair by end-joining pathways (Table 1). The majority of end-joining reporters are conceptually similar and contain an intact GFP gene that is not expressed due to an upstream inhibitory sequence element, like an out-of-frame start codon or a second gene with a stop codon (Figure 1D). DSBs are generated at two nuclease target sites flanking this inhibitory sequence, and fusion of the distal DSB-ends by end-joining repair will excise the inhibitory element and permit GFP expression. Some end-joining reporters generate ligation-compatible distal DSB-ends (see Figure 1D), and will therefore measure the collective frequency of error-free c-NHEJ, mutagenic c-NHEJ, and MMEJ. Other end-joining reporters are limited to the detection of mutagenic end-joining as their distal DSB-ends have non-compatible overhangs (Figure 1D; Table 1). In both reporter types, however, the contribution of each individual end-joining pathway to DSB-repair cannot be distinguished based on GFP expression, although it can be revealed by sequence analysis of the repair junction (Bennardo et al., 2008).

In contrast to these generic end-joining reporters, the pMX-INV reporter specifically measures c-NHEJ (Bredemeyer et al., 2006). It is based on the process of VDJ recombination that occurs in antigen-receptor genes during lymphocyte maturation (Bassing et al., 2002). The pMX-INV reporter is introduced in mouse progenitor B-cells that express viral Abl kinase and are arrested in G1 by Alb kinase inhibitors. This induces expression of the RAG nucleases that cleave adjacent to recombination signals in the reporter to excise an antisense GFP gene (Figure 1E). Subsequently, the GFP gene is inverted and re-ligated during a VDJ recombination-like reaction, which puts it behind the LTR promoter and results in GFP expression. As VDJ recombination is strictly dependent on c-NHEJ repair of the RAG-induced DSBs (Helmink and Sleckman, 2012), GFP expression from pMX-INV is a direct measure for c-NHEJ activity. More recently the EJ7-GFP reporter was developed, which also specifically quantifies repair by c-NHEJ, but resembles the other end-joining reporters rather than pMX-INV, and, unlike pMX-INV, can be used in any genetically modifiable cell-line. It contains a GFP gene with an intragenic 46 basepair (bp) spacer sequence that can be removed by targeting Cas9 precisely to the edges, and which results in an intact GFP sequence if the distal ends are joined by error-free c-NHEJ (Figure 1F; Bhargava et al., 2018).

Finally, the Stark lab published two reporters that were designed to monitor MMEJ. The EJ2-GFP reporter contains two regions with 8 nucleotides microhomology flanking an I-*Sce*I site. Repair of the I-*Sce*I-induced DSB by MMEJ will remove a stop codon and put GFP in frame with an upstream NLS-tag sequence (Figure 1G; Bennardo et al., 2008). Notably, sequence analysis of repair junctions from GFP positive cells revealed that 10% of the repair products contained a deletion without microhomology flanking the break, indicating that microhomology-independent DSB-repair of EJ2-GFP can also result in GFP expression. Furthermore, several EJ7-GFP variants were generated containing microhomology (Bhargava et al., 2018). One variant, with 4 nucleotides microhomology located inward from the DSB-edge, is a bona fide MMEJ reporter, as GFP expression was strongly dependent on the end-resection factor CtIP, and the MMEJ-factor PolΘ (Bhargava et al., 2018).

## Multi-Pathway DSB-Repair Reporters

HR, SSA and end-joining are connected in a DSB-repair signaling network, such that loss of a pathway is compensated for by enhanced engagement of one or more of the remaining pathways (Scully et al., 2019). To study these inter-pathway dynamics, reporter systems have been developed that can quantify repair by more than one pathway (Table 1). Many multi-pathway reporters combine elements from published single-pathway reporter systems, and contain two or more genes encoding different fluorescent proteins to distinguish between the repair pathways. Rather than providing a detailed explanation of each individual multi-pathway reporter, we will discuss a few to highlight the major design concepts, and refer the reader to the references listed in Table 1 for details.

The majority of multi-pathway reporters were developed to simultaneously monitor end-joining and HR (Table 1). This was often achieved by combining a genomic end-joining reporter with an ectopic repair template. For example, the Traffic Light Reporter (TLR) contains a GFP gene disrupted by an internal I-*Sce*I site, followed by an mCherry gene with a reading frame shifted 2 bp compared to that of GFP (Certo et al., 2011). Formation of any 2 bp frameshift-causing mutation at the I-*Sce*I target site, predominantly generated by mutagenic c-NHEJ, will result in mCherry expression. HR can be analyzed by GFP expression, which is caused by gene conversion between the I-*Sce*I site-containing GFP gene and a truncated GFP gene present on an ectopically provided repair template.

In several other multi-pathway reporters gene conversion causes a color-switch, i.e., a change from expression of one fluorescent protein to another. A particularly practical and widely used HR-dependent color switch is GFP to BFP conversion, or the other way around (Glaser et al., 2016). The BFP and GFP genes are completely homologous with exception of two amino acids that determine their fluorescent properties, and can therefore function as reciprocal repair templates without the need for additional homologous sequences. BFP to GFP conversion is also used as read-out for HR in the DSB-Spectrum reporters (van de Kooij et al., 2021). Conveniently, no ectopic repair template is required in these multi-pathway reporter systems because the truncated GFP repair template is located on the same construct, downstream of the BFP gene that is targeted by Cas9.

Several dual pathway reporters monitor sub-pathways rather than any of the four major DSB-repair pathways. A recent example of such a reporter is the CDDR (two cut variant), which contains a functional mCherry gene inserted into a split, and thus non-functional, GFP gene (Eki et al., 2020). Cas9 is targeted to sites flanking the mCherry gene such that distal error-free c-NHEJ reconstitutes the GFP gene, and deletes the mCherry gene. This can be distinguished from mutagenic end-joining, which results in mCherry loss without GFP expression.

Finally, whereas there are many dual-pathway reporters, only two systems can monitor three pathways simultaneously. SSA-TLR is a variant of the TLR that is flanked by truncated iRFP genes, which can be joined by SSA to form an intact gene (Kuhar et al., 2016). This system thus reports on 2 bp frameshift inducing c-NHEJ, HR with an ectopic repair template, and SSA. The more recently developed DSB-Spectrum_V3 consists of an intact BFP gene that is targeted by Cas9, and can be disrupted by end-joining mediated mutagenesis. Loss of BFP expression is thus a measure for mutagenic end-joining in general, but primarily for mutagenic c-NHEJ, as indicated by sequence analysis (van de Kooij et al., 2021). Furthermore, the reporter contains a truncated GFP gene that can be used to convert BFP to GFP as a measurement for HR (van de Kooij et al., 2021). These homologous elements can also anneal during DSB-repair by SSA, resulting in the removal of a functional mCherry gene that separates the two. As such, a single reporter can be used to simultaneously measure three DSB-repair pathways.

## Considerations for Using DSB-Repair Reporters

There is substantial variation within the reporter repertoire, and one reporter might be more suited to specific research needs than the other. A first consideration is whether to use single-pathway or multi-pathway reporter systems. An advantage of single-pathway systems is their simplicity and, at least for many of them, their extensive validation by widespread use for many years. However, with similar research efforts, multi-pathway reporters generate a more comprehensive view of DSB-repair pathway activity. When studying DBS-repair factors, for instance, such reporters can immediately reveal whether a factor functions in one or multiple pathways, the latter of which can also indicate at which node in the DSB-repair network it acts.

A second consideration is to use either reporters that require ectopic HR repair templates, or repeat-containing reporters that carry the template embedded within the construct. Glaser *et al.* used a single-stranded oligo as template in their GFP to BFP conversion reporter (Glaser et al., 2016), which has been demonstrated to mediate gene conversion by a mechanism that diverges from canonical HR (Bothmer et al., 2017; Richardson et al., 2018). The other templated reporters use double-stranded repair templates, which might be copied in an HR-dependent process. However, unlike the sister chromatid, these ectopic repair templates are present in high copy number, exist throughout the cell cycle, and lack proximity to the broken chromosome. In repeat-containing reporters, the template is on the sister chromatid in S/G2. However, it is also present in G1 and could theoretically be used as a donor during intrachromosomal gene conversion, in contrast to HR at endogenous loci. Although these non-physiological HR-events cannot be completely excluded, early studies on DR-GFP-like reporters indicated them to be rare (Johnson and Jasin, 2000). Moreover, more recently it was shown that all detected gene conversion events with a novel repeat-containing reporter occurred in S-phase (Chen et al., 2019). Therefore, repeat-containing HR-reporters more accurately reflect HR-repair at endogenous genomic loci and may be preferred when studying fundamentals of DSB-repair, whereas templated reporters can be useful when studying genome editing approaches.

A third consideration is that some reporters, in particular end-joining reporters, might not be very specific but actually measure the collective frequency of repair by multiple pathways, as explained above. More specific detection of repair by a single pathway is in most cases preferable. However, it should be taken into account that too high specificity can come at the cost of low frequency. This is, for example, the case in the RFP-SCR reporter, which can distinguish between gene conversion sub-pathways. The frequency of cells undergoing long-tract gene conversion is consistently less than 0.1%, requiring analysis of large cell populations for reliable quantification (Chandramouly et al., 2013).

Fourth, reporters are designed to detect DSB-repair either by loss or by induction of marker gene expression. The latter confers specificity because the expression is strictly dependent on a defined change in the reporter sequence (Figure 1). This could, however, result in an underestimation of pathway usage because not all repair by the assayed pathway necessarily generates that defined sequence change. For example, mCherry expression in the TLR is dependent on generation of 2 bp frameshifts, and it therefore measures only a fraction of mutagenic c-NHEJ (Certo et al., 2011; Kuhar et al., 2016). Loss of marker gene expression, on the other hand, is theoretically less specific because it could be caused by other mutagenic repair pathways than the one measured. Nevertheless, these reporters are more inclusive because they lack the requirement for a unique mutagenic event, and their specificity can easily be validated by sequencing and genetic interrogation.

Finally, even though reporter constructs have proven extremely useful they do have limitations. First, the DSB is generated in a specific sequence context, which can impact repair pathway employment (Shen et al., 2018; Allen et al., 2019). Second, the DSB in reporter constructs is generated by efficient nucleases that keep cutting as long as they are expressed and the target site is intact. As a consequence, the phenotype analyzed might be the result of re-iterative rounds of error-free repair followed by one mutagenic repair event. This might result in an overestimation of the frequency of mutagenic repair. Moreover, the persistence of a constantly regenerated DSB might in specific cases affect pathway choice (Bennardo et al., 2009). Third, the nucleases will most likely cut both sister chromatids, which disables HR and makes the cell more reliant on alternative pathways like SSA or MMEJ. This should be taken into account when using, for example, SSA-reporters. It is, however, not an issue when using HR reporters, because the provided template lacks the nuclease target site. Altogether, these limitations should be considered for correct interpretation of reporter assays. However, they do not prevent the generation of insightful data, as proven by a large body of published DSB-repair literature in which results obtained with reporter assays were validated using orthogonal techniques.

## Future Developments of DSB-Repair Reporters

The advent of targetable nucleases like Cas9 has spurred the construction of novel reporter systems (Table 1). It has enhanced the flexibility in reporter design because it negates the requirement for a specific I-*Sce*I nuclease target sequence. Moreover, it has also expanded the nuclease repertoire with a variety of blunt-cutting enzymes, the staggered cutting Cas12a/Cpf1, and Cas9 nickase variants that can be targeted to either strand (Komor et al., 2017). Therefore, different DSB-ends can be generated, which has been shown to affect pathway choice (Bothmer et al., 2017; Schimmel et al., 2017). Given these enhanced design possibilities, a four-pathway reporter system measuring the frequency of all major DSB-repair pathways is within reach.

In addition to the development of new reporters, genome editing tools will facilitate targeted integration of reporter constructs. This will allow for comparison of repair pathway usage between genomic loci, for example, in hetero- and euchromatic regions, which is an active area of investigation (Caron et al., 2021; Schep et al., 2021). Interestingly, Cas9-based tools have been developed to modify the chromatin at target loci (Goell and Hilton, 2021). These could be used in combination with reporters to study the effect of specific chromatin marks on DSB-repair pathway choice. Importantly, ongoing research efforts are aimed at developing methods to rapidly activate and de-activate Cas9 (Liu et al., 2020; Marino et al., 2020), so that complete temporal control over Cas9 activity will be possible in the near future, thus eliminating the current problem of re-iterative cutting of reporters. Finally, reporters are ideal tools for pooled high-throughput screening, because repair phenotypes can easily be selected by FACS. Reporter screens have been done using siRNAs, but these screens have been hampered by the strong tendency of siRNAs to silence Rad51 expression as an off-target effect (Adamson et al., 2012; Howard et al., 2015). CRISPR-based screens are generally less affected by off-target editing, and the first insightful CRISPRi reporter screens have already been published (Richardson et al., 2018; Wienert et al., 2020).

In conclusion, DSB-repair reporters have evolved from designated constructs to study HR, to complex multicolor tools that can measure repair by two or three pathways in one assay. This evolution is expected to continue, driven by Cas9-technology, ensuring that reporters will remain an essential element in the DSB-repair toolkit, as they have been for multiple decades.
